# Tissue Models for *Neisseria gonorrhoeae* Research—From 2D to 3D

**DOI:** 10.3389/fcimb.2022.840122

**Published:** 2022-02-11

**Authors:** Motaharehsadat Heydarian, Eva Rühl, Ravisha Rawal, Vera Kozjak-Pavlovic

**Affiliations:** Chair of Microbiology, Biocenter, University of Würzburg, Würzburg, Germany

**Keywords:** *Neisseria gonorrhoeae*, *in vivo*, *in vitro*, ex vivo, biomimetic tissue models

## Abstract

*Neisseria gonorrhoeae* is a human-specific pathogen that causes gonorrhea, the second most common sexually transmitted infection worldwide. Disease progression, drug discovery, and basic host-pathogen interactions are studied using different approaches, which rely on models ranging from 2D cell culture to complex 3D tissues and animals. In this review, we discuss the models used in *N. gonorrhoeae* research. We address both *in vivo* (animal) and *in vitro* cell culture models, discussing the pros and cons of each and outlining the recent advancements in the field of three-dimensional tissue models. From simple 2D monoculture to complex advanced 3D tissue models, we provide an overview of the relevant methodology and its application. Finally, we discuss future directions in the exciting field of 3D tissue models and how they can be applied for studying the interaction of *N. gonorrhoeae* with host cells under conditions closely resembling those found at the native sites of infection.

## Introduction

The sexually transmitted disease (STD) gonorrhea caused by *N. gonorrhoeae* is the second most common STD which causes around 86.9 million new infections annually around the world, as estimated for 2016 (Rowley et al., 2019). The rapid increase in antibiotic resistance makes gonorrhea a serious threat to public health worldwide (Unemo and Shafer, 2011; Rubin et al., 2020). The mucosal surface of the female cervix and male urethra, anorectal, pharyngeal, and conjunctival areas are the most common sites of *N. gonorrhoeae* infection (Miller, 2006). *N. gonorrhoeae* can pass into the deeper tissue layers and reach the bloodstream, causing systemic disseminated gonococcal infections (DGI) in 0.5% to 3% of patients, which leads to endocarditis, meningitis, peri hepatitis, and permanent joint damage (Knapp and Holmes, 1975; Cannon et al., 1983; Kerle et al., 1992; Hansen et al., 2014).

**Figure 1:**
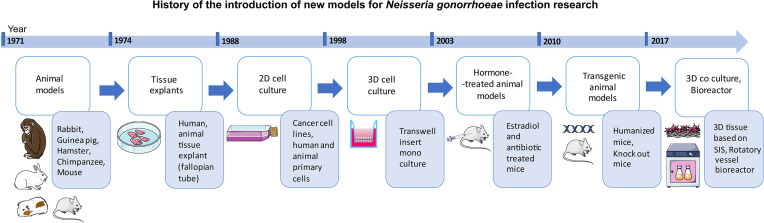
Schematic representation of the history of the introduction of new models into the field of *Neisseria gonorrhoeae* infection research.

History of gonococcal infection research traces back to the use of animal models of rabbits, guinea pigs, hamsters, mice, and chimpanzees (Miller et al., 1945; Lucas et al., 1971; Arko, 1972) (**Figure 1**). Finding a proper model for gonorrhea research, however, has always been a challenge since *N. gonorrhoeae* is a human-specific pathogen. Several *in vitro* cell models have been established using various cell sources including cell lines (cancer and immortalized) and primary cells (Shaw and Falkow, 1988; Harvey et al., 1997; Giardina et al., 1998; Scheuerpflug et al., 1999; Christodoulides et al., 2000; Edwards et al., 2000; Hopper et al., 2000; Fichorova et al., 2002). Apart from animal models, a variety of approaches have been used to recreate the 3D structure of the site of infection as well as pathogen penetration and immune cell transmigration. These have included: Transwell^®^ insert technology (Merz et al., 1996; Edwards et al., 2000; Stevens et al., 2018), decellularized scaffolds (Heydarian et al., 2019), as well as organ cultures derived from various sites within the urogenital tract (Carney and Taylor-Robinson, 1973; Tjia et al., 1988; Mosleh et al., 1997; Timmerman et al., 2005; Yu et al., 2019). The present review focuses on the established *in vivo* and *in vitro* tissue models used in investigations of the infection caused by gonococci. The recent advances in gonorrhea disease research using animal and *in vitro/ex vivo* tissue models are also reviewed. Finally, we discuss the main challenges and obstacles, followed by future perspectives in the field of models used for studying gonococcal infection.

## Animal Models

Human male volunteers have been used as experimental subjects (Cohen et al., 1994; Hobbs et al., 2013) and have helped understand the function of various virulence factors of gonococci as well as the immune response to infection, which can support vaccine research [reviewed in (Hobbs et al., 2011)]. Studies using human-derived source material have provided important insights into the pathogenesis of gonococcal infection in men (Ward and Watt, 1972; Apicella et al., 1996). Nevertheless, due to the ethical limitation, in-depth research of the mechanism of gonococcal infection has mostly relied on animal models, which provide a great tool for studying *N. gonorrhoeae* infection and the corresponding immune response, due to the presence of the intact immune system (Lucas et al., 1971; Arko, 1972; Arko, 1974).

Several non-human species studies, which used rabbits (Miller et al., 1945), guinea pigs (Novotny et al., 1978; Wong et al., 1979), hamsters (Arko, 1972), mice (Streeter and Corbeil, 1981; Johnson et al., 1989), chimpanzees (Lucas et al., 1971; Kraus et al., 1975), or recently greater wax moth larvae (Dijokaite et al., 2021) (**Table 1**) have been performed to study the disease development and for drug discovery. In 1990, for the first time, Taylor-Robinson and colleagues performed successful colonization of mice with *N. gonorrhoeae* using estradiol-treated germ-free female mice (Taylor-Robinson et al., 1990). The 17β-estradiol treatment promotes the long-term colonization of *N. gonorrhoeae* by the expansion of the estrus phase. However, as during this treatment the overgrowth of commensal flora of mice can prevent the attachment of the gonococci, usage of germ-free mice or antibiotics enhances the success of the colonization by *N. gonorrhoeae* (Jerse, 1999; Hobbs et al., 2013; Kim et al., 2019). Depending on the type of estradiol treatment, the gonococci can persist in the murine genital tract from 10 to as long as 40 days, but the success of colonization and host response are dependent on the mouse strain used [reviewed in (Jerse et al., 2011)]. Usually, the infection is performed by intravaginal inoculation (Johnson et al., 1989; Taylor-Robinson et al., 1990; Jerse, 1999), or, for the modeling of the upper reproductive tract infection, by transcervical inoculation (Corbeil et al., 1978; Connolly et al., 2021). These mouse models have contributed to the better understanding of the immune response to gonococci (Song et al., 2008; Feinen et al., 2010), and the role of the MtrC–MtrD–MtrE efflux pump system in infection (Jerse et al., 2003; Warner et al., 2007) to name the few examples.

**Table 1:** Selected animal models used for studying *N. gonorrhoeae* infection.

Animal Model	Key findings	Reference
Rabbit	*N. gonorrhoeae* invade and multiply in the lens and ciliary bodies in the eye of infected rabbits.	(Miller et al., 1945)
Chimpanzee	Introducing chimpanzee as a model of choice for studying *N. gonorrhoeae* infection.	(Lucas et al., 1971)
Rabbit, guinea pig, hamster, mouse	Infecting various laboratory animals with *N. gonorrhoeae*; introducing guinea pig as the most relevant model for studying the immune response to *N. gonorrhoeae.*	(Arko, 1972) (Arko, 1974)
C3H, CBA, BALB/c, TO, and ICR mice	Resistance of mice to gonococcal infection.	(Johnson et al., 1989)
Estradiol treated germ-free female BALB/c mice	The first evidence of sustained mucosal colonization in mice upon estradiol treatment.	(Taylor-Robinson et al., 1990)
Estradiol treated female BALB/c mice	The role of mtrCDE-encoded and farAB-encoded efflux pump systems in *N. gonorrhoeae* infection in mice.	(Jerse et al., 2003) (Warner et al., 2007)
Knockout C57BL/6 BG mice and THP1 cell line	Investigation of the immune response to *N. gonorrhoeae* by focusing on IL-17 and Th17 cells induction upon infection.	(Feinen et al., 2010)
hCEACAM1 FVB and C57BL/6 BG transgenic mice	Establishment of human CEACAM1 transgenic mouse model.	(Gu et al., 2010) (Li et al., 2011)
C57BL/6 mice transgenic for human CEA	Bacteria colonize the urogenital tract of CEA transgenic mice by suppressing the exfoliation of mucosal cells.	(Muenzner et al., 2010) (Muenzner and Hauck, 2020)
Human, estradiol treated female BALB/c mice	Study of the role of PEA-decorated gonococcal lipid A in competitive infections in female mice and male volunteers.	(Hobbs et al., 2013)
Transgenic mice (hCEACAM1, CEABAC2)	The role of individual Opa-CEACAM interactions in uncomplicated lower genital tract infections in comparison to pelvic inflammatory disease.	(Islam et al., 2018)
Human CD34+ stem cell transplanted NSG mice	*N. gonorrhoeae* co-infection with HIV increases the female genital tract viral shedding.	(Xu et al., 2018)
hCEACAM1, CEABAC2 and hCEACAM5 transgenic mice	Transcriptional analysis of infected mice; mice diestrus uterine infection shows higher type-1 interferon induction.	(Francis et al., 2018)
Estradiol treated female BALB/c mice	Commensal species of *Neisseria* kill Ngo through a mechanism based on genetic competence and DNA methylation state.	(Kim et al., 2019)
An invertebrate *Galleria mellonella* greater wax moth larvae	Testing of the anti-gonococcal properties of antibiotics and novel antimicrobials.	(Dijokaite et al., 2021)

Certain similarities between the physiology of the human and mouse reproductive tract exist. The average vaginal pH in women is below 4.5, whereas the cervix has a pH in the range of 6.5 to 7.5 (Ng et al., 2018). In comparison, the average vaginal pH of 17β-estradiol-treated mice that are susceptible to *N. gonorrhoeae* infection is 6.6, similar to the pH of the human cervix, which is the primary site of infection for humans (Jerse, 1999; Muench et al., 2009). However, there are also some anatomical and endocrinological differences between mice and men [reviewed in (Cunha et al., 2019)]. For instance, the vaginal flora in mice and humans are dissimilar (Noguchi et al., 2003). Another reason that the mouse model fails to appropriately represent human physiology is the lack of certain host-specific receptors, which are the binding target of different virulence factors of *N. gonorrhoeae* (Jerse et al., 2011; Quillin and Seifert, 2018).

To address this issue, a variety of humanized transgenic mice has been generated. These express different receptors of gonococcal virulence factors, such as human (h)CEACAM receptors, to increase the similarity of the mouse model to humans (Eades-Perner et al., 1994; Gu et al., 2010; Muenzner et al., 2010; Li et al., 2011). Such models were used to show that CEACAM-binding gonococci colonize the urogenital tract of genetically modified mice by suppressing the exfoliation of mucosal cells (Muenzner et al., 2010; Muenzner and Hauck, 2020). Transgenic mice for hCEACAM1, hCEACAM5, and CEABAC2 were also successfully used to reproduce the differential expression of CEACAM1 and CEACAM5 on the surface of the epithelial cell monolayer of the upper and lower female genital tract. This enabled the studies of the effect of Opa-CEACAM mediated interactions during the gonococcal infection along the female genital tract (Islam et al., 2018). Another example of humanized mice models is the engrafting of the immunodeficient NSG mice with human CD34+ hematopoietic stem cells. These mice were used to study *N. gonorrhoeae* and HIV co-infection (Xu et al., 2018).

Although transgenic animals are promising models for studying the early stages of *N. gonorrhoeae* infection (Wang et al., 2008; Gu et al., 2010; Francis et al., 2018), they cannot fully recapitulate all the features of gonorrhea in humans (Wang et al., 2017a; Quillin and Seifert, 2018; Stevens and Criss, 2018). Nevertheless, only a few non-transgenic animal models such as chimpanzees were successfully infected by *N. gonorrhoeae* and showed a greater resemblance of the symptoms and host reaction (Kraus et al., 1975). However, the application of primate models is limited due to the low availability and high maintenance costs (DiGiacomo et al., 1977; Jerse, 1999). In conclusion, the most abundant animal models used in the field of gonorrhea research are estradiol-treated wild-type and transgenic mice expressing human receptors (**Table 1**).

## *In Vitro* 2D Cell Models

Over the past fifty years, a great majority of research on *N. gonorrhoeae* pathogenesis has been conducted using 2D cell culture of cell lines and primary cells (**Table 2**). In these *in vitro* models, cells are normally cultured on cell culture well plates, with and without coating with extracellular matrix (ECM) proteins such as collagen and Matrigel (Harvey et al., 1997; Christodoulides et al., 2000; Simons et al., 2005). Commonly used epithelial cell lines include HeLa cells (Brodeur et al., 1977; Gray-Owen et al., 1997; Naumann et al., 1997), human endometrial adenocarcinoma cells (HEC-1-B) (Shaw and Falkow, 1988), epidermoid carcinoma cervix cells ME180 (Naumann et al., 1997), human colorectal carcinoma cells (T84) (Merz et al., 1996), human conjunctiva epithelial cells (Chang) (Grassmé et al., 1996), and human Asian endometrial adenocarcinoma cells (Ishikawa) (Rodríguez-Tirado et al., 2012).

**Table 2:** Selected *in vitro* 2D cell culture models for studying *N. gonorrhoeae* infection.

Cells	Key Findings	Reference
Human sperm	The role of pilus in the attachment of gonococci to human sperm.	(James-Holmquest et al., 1974)
HeLa, HEp-2	Interaction of gonococci with tissue culture cells.	(Brodeur et al., 1977)
HEC-1-B cell line	The HEC-1-B cell line was introduced as a model for studying the invasion of *N. gonorrhoeae.*	(Shaw and Falkow, 1988)
Corneal epithelial cells, erythrocytes	The requirement of interaction between PiLE and PiLC for pilus mediated adherence of *N. gonorrhoeae* to the host.	(Rudel et al., 1992)
Chang cell line	The role of Opa in rearrangements of the epithelial cell actin cytoskeleton.	(Grassmé et al., 1996)
T84 cell line	Traversal of polarized epithelium by *N. gonorrhoeae*. The polarization induced by growing on Transwell^®^ inserts.	(Merz et al., 1996)
HeLa, CHO, HUVECs	Investigation of Opa^-^ CD66 interactions and cellular response to *N. gonorrhoeae.*	(Gray-Owen et al., 1997)
HeLa, ME180, HaCaT keratinocytes	Activation of NF-κB and the transcriptional activation of inflammatory cytokine genes upon infection of epithelial cells.	(Naumann et al., 1997)
Primary urethral epithelial cell	Development of primary male urethral epithelial cell culture method for studying gonococcal infection.	(Harvey et al., 1997)
Primary human endometrial cells	The role of pili and Opa proteins in interactions of *N. gonorrhoeae* with endometrial cell.	(Christodoulides et al., 2000)
Primary ecto- and endo-cervical cells	Membrane ruffles appear to be induced in response to gonococci. Culturing in 2D with transfer to Transwell^®^ inserts.	(Edwards et al., 2000)
Endocervical (End1), Ectocervical (Ect1), vaginal (Vk2), and endothelial (HMEC1) cells	Toll-Like Receptor 4-mediated signaling during the immune response to the *N. gonorrhoeae* infection.	(Fichorova et al., 2002)
THUEC immortalized primary urethral cells	Study of the inflammatory cytokine response to gonococcal infection.	(Harvey et al., 2002)
Fallopian tube epithelial cells	Gonococcal infection inhibits TNFα-induced apoptosis.	(Morales et al., 2006)
THCEC immortalized primary cervical cells	Study of the mechanism of gonococcal biofilm formation.	(Falsetta et al., 2009)
Ishikawa cell line	Cell junction disruption in human genital epithelial cells is independent of Opa and Pili.	(Rodríguez-Tirado et al., 2012)

In 1988, Shaw and Falkow reported the first *in vitro* monoculture model based on HEC-1-B cells for studying the invasion of *N. gonorrhoeae*. Here, the authors were able to document the invasion of gonococci, which was inhibited by cytohalasin D and was not affected by the state of piliation of the bacteria (Shaw and Falkow, 1988). *N. gonorrhoeae* can also be transmitted from infected mothers to their infants during birth, causing neonatal blindness (Rees et al., 1977; Woods, 2005; Quillin and Seifert, 2018). Therefore, adherence and invasion of gonococci were studied in the cultured human corneal epithelial cells, showing that PilC plays a central role in pilus-mediated adherence of the bacteria (Rudel et al., 1992). Human Chang cells were also used for studying the Opa-mediated invasion of *N. gonorrhoeae* (Grassmé et al., 1996). It is important to note that the Chang cell line, originally thought to be derived from human conjunctiva, was later shown to have been established through contamination with HeLa cells (Lavappa, 1978; Capes-Davis et al., 2010) similar to the Hep-2 cell line (Brodeur et al., 1977). Nevertheless, Chang cells are an important tool for studying the interaction of gonococci with epithelial cells if one is not exploring tissue type-specific effects. For example, the Chang cell line has been used by our department to show the necessity of neutral sphingomyelinase 2 activity for the PorB_IA_-dependent invasion of *N. gonorrhoeae* under phosphate-free conditions (Faulstich et al., 2015).

Despite the advantages of immortalized cell lines, which include availability, easy handling, and expansion (Pan et al., 2009), they have their drawbacks, such as being derived from tumor tissues or showing significant changes from the original cell type caused by immortalization and prolonged cultivation. Alternatively, different *in vitro* models using primary cells or immortalized primary cells have been established. These include early attempts using human sperm (James-Holmquest et al., 1974) or the cells derived from the urethral epithelium (Harvey et al., 1997; Giardina et al., 1998; Zenni et al., 2000; Harvey et al., 2001; Edwards and Apicella, 2005), as well as the immortalized primary urethral cells (Harvey et al., 2002). Primary urethral epithelial cells were mostly used to provide a model system similar to the native tissues for studying the attachment and invasion of gonococci (Harvey et al., 2001), or cytokine response to infection (Harvey et al., 2002) but in some cases were used to confirm the validity of the observations made in tumor cell lines (Giardina et al., 1998). Primary cells derived from different parts of the female reproductive tract were also used in various studies. Among them are those of cervix (Edwards et al., 2000; Swanson et al., 2001; Edwards et al., 2002; Steichen et al., 2008; Edwards, 2010; Jennings et al., 2011), endometrium (Christodoulides et al., 2000; Timmerman et al., 2005), and fallopian tube (Swanson et al., 2001; Maisey et al., 2003; Morales et al., 2006; Reyes et al., 2007). Additionally, immortalized human cervical epithelial cells were introduced in 2009 and used in studies of gonococcal biofilm formation (Falsetta et al., 2009) as well as studies of *N. gonorrhoeae* interaction with cell surface glycans (Semchenko et al., 2019). Primary cell culture systems proved to be useful for the *N. gonorrhoeae* host cell interaction studies where the role of certain receptors was investigated or where the induction of immune response was studied, as well as for the ultrastructural analysis of infected cells. For example, some cancer cell lines such as HEC-1-B and HeLa were shown not to express CEACAM molecules, whereas primary cervical cells, but also ME180 cell line, did (Swanson et al., 2001). Primary cells are also a model of choice when various signaling or cell death pathways are investigated or in gene expression studies (Reyes et al., 2007), because tumor cell lines might be too divergent from the original tissues for these purposes. From the technical view, the fact that most primary cells can be passaged for only a few passages represents a significant drawback. The introduction of immortalized primary cells can offer a solution to this problem while providing a model system that is still closer to the native tissue than tumor cell lines.

Apart from colonizing epithelia, *N. gonorrhoeae* can cause systemic infections, due to its ability to reach deeper tissue layers and blood stream. During its traversal across tissues, it encounters a variety of non-epithelial cells; therefore, cells residing beneath the epithelium such as fibroblasts and endothelial cells have also been used to investigate host-pathogen interaction (Scheuerpflug et al., 1999; Fichorova et al., 2002). Fibroblasts are responsible for the structure and shape of the connective tissue by producing and secreting the ECM components such as collagen, glycosaminoglycans (GAGs), and proteoglycans (Le and Brown, 2012). In addition to epithelial cells, which are capable of innate responses to infection, including the production of complement components, prostaglandins, and cytokines, and in some cases are hormone-responsive (Schleimer et al., 2007; Hickey et al., 2011), fibroblasts also play a role in pathological and immune responses by producing and responding to various cytokines (Delves and Roitt, 1998; LeBleu and Neilson, 2020). In particular, cervical fibroblasts can change their biomechanical properties in response to different stimuli such as pro-inflammatory cytokines (e.g. IL-1β) and hormones (e.g. progesterone), resulting in differential remodeling of the ECM (Shukla et al., 2018). Studying *N. gonorrhoeae* infection in a monoculture model of murine fibroblasts showed an increase in the number of viable bacteria recovered from the infected cells up to 48 hours post-infection. This effect was not inhibited by washing, indicating that *N. gonorrhoeae* was attached to and/or internalized by fibroblast cells (Waitkins and Flynn, 1973). Furthermore, it has been shown that the activation of the phosphatidylcholine-specific phospholipase C (PC-PLC) and acid sphingomyelinase (ASM) by *N. gonorrhoeae* is crucial for the pathogen entry into the human fibroblasts (Grassmé et al., 1997).

Infection with *N. gonorrhoeae* triggers a potent innate immune response, which involves neutrophils (also known as polymorphonuclear leukocytes or PMNs). Several reports showed the presence of viable bacteria in gonorrheal exudates, indicating that PMNs are not able to completely clear gonococci (Johnson and Criss, 2011). Various approaches were used to study the *N. gonorrhoeae*-neutrophil interaction (**Table 3**). A variety of neutrophil-like cell lines, such as differentiated human leukemia (HL-60) (Bauer et al., 1999; Pantelic et al., 2004), and human monocytic (THP-1) have been developed. These cell lines retain most of the features of neutrophils. For example, HL-60 cells induced by retinoic acid express CEACAM1 and can phagocyte gonococci (Pantelic et al., 2004) and have been shown to be an appropriate model for *N. gonorrhoeae*-PMN interaction (Chen and Seifert, 2011). However, HL-60 cells also lack specific granules and fail to show the antimicrobial activity associated with primary cells (Le Cabec et al., 1997). Human neutrophils isolated from blood as primary cell models helped understand the role of virulence factors in pathogen invasion and disruption of immune cell functions (Watt, 1970; Thongthai and Sawyer, 1973; Simons et al., 2005; Stohl et al., 2013; Johnson et al., 2015; Reimer et al., 2016). They were used to show that gonococci are resistant to engulfment by neutrophils and can multiply inside them (Simons et al., 2005), as well as that they can inhibit neutrophil apoptosis and activate the secretion of proinflammatory cytokines (Chen and Seifert, 2011).

**Table 3:** Selected *in vitro* models of immune cells for studying *N. gonorrhoeae* infection.

Cells	Key Findings	Reference
Human and guinea pig primary neutrophils	Bactericidal activity of PMNs on gonococci.	(Watt, 1970)
Human and rabbit primary neutrophils	Relation between colony morphology and resistance to phagocytosis.	(Thongthai and Sawyer, 1973)
Human monocyte-derived macrophages	Neisserial porin can arrest phagosome maturation within macrophages.	(Mosleh et al., 1998)
Human CD4^+^ T lymphocytes	Gonococcal Opa proteins can bind to primary CD4^+^ T lymphocytes and suppress their activation and proliferation.	(Boulton and Gray-Owen, 2002)
HL-60 cell line	Retinoic acid treated HL-60 cells express CEACAM1 and can phagocytose Opa-expressing gonococci.	(Pantelic et al., 2004)
Human primary neutrophils	Resistance and replication of *N. gonorrhoeae* inside neutrophils.	(Simons et al., 2005)
Immature human dendritic cells	Gonococci activate dendritic cells through TLR2, enhancing HIV-1 infection of these cells.	(Zhang et al., 2005)
Chicken DT40 B cells, B cell-enriched peripheral blood mononuclear cells	*N. gonorrhoeae* kills CEACAM1 (CD66a)-expressing human B cells, inhibiting antibody production.	(Pantelic et al., 2005)
Primary human T cells, Jurkat-CEACAM1 CD4^+^ T cell line	*Neisseria gonorrhoeae* suppresses CD4^+^ T lymphocyte activation.	(Lee et al., 2007; Lee et al., 2008)
Human dendritic cells	*N. gonorrhoeae* lipooligosaccharide variation directs dendritic cell-induced T helper responses.	(van Vliet et al., 2009)
HL-60 cell line and human primary neutrophils	*N. gonorrhoeae* actively inhibits apoptosis and activates NF-κB signaling in neutrophils.	(Chen and Seifert, 2011)
Mouse bone marrow-derived dendritic cells and T lymphocytes	*N. gonorrhoeae*-exposed dendritic cells fail to elicit antigen-induced CD4^+^ T lymphocyte proliferation.	(Zhu et al., 2012)
Mouse spleen T lymphocytes	*N. gonorrhoeae* suppresses Th1/Th2-mediated adaptive immune response through the TGF-β-dependent mechanism.	(Liu et al., 2012)
Human B lymphocytes	Gonococci interact with human IgM memory B cells, activating them and eliciting a broad, T cells-independent Ig response.	(So et al., 2012)
Mouse RAW 264.7 macrophage cell line	*N. gonorrhoeae* induces a tolerogenic phenotype in macrophages to induce immune response evasion.	(Escobar et al., 2013)
Human monocyte-derived macrophages	*N. gonorrhoeae*-treated macrophages are unable to induce the proliferation of human T cells.	(Ortiz et al., 2015)
U937 and THP-1 cell lines, monocyte-derived macrophages	Interaction of *N. gonorrhoeae* and macrophages, and the role of macrophages as a niche for replication of *N. gonorrhoeae.*	(Château and Seifert, 2016)
Human monocyte-derived macrophages	*N. gonorrhoeae* induces inflammatory pyroptosis in human macrophages in connection to intracellular LOS.	(Ritter and Genco, 2018)
U937 and primary human peripheral monocytic cells	*N*. *gonorrhoeae* occupy distinct subcellular niches when colonizing macrophages.	(Ivanov et al., 2021)

The role of other immune cells in gonococcal infection has also been addressed in various studies (**Table 3**). Macrophage models include murine RAW 264.7 and human U937 and THP-1 cell lines, as well as human monocyte-derived primary macrophages (MDMs), which can be differentiated *in vitro* towards different phenotypes, M1 or M2 [reviewed in (Escobar et al., 2018)]. U937, THP-1, and MDMs have been used to address the interaction of gonococci with macrophages and show that gonococcal porin can arrest phagosome maturation within the macrophages (Mosleh et al., 1998), which might be connected to the further observations that macrophages represent a niche for gonococcal replication (Château and Seifert, 2016), where bacteria occupy distinct subcellular niches when colonizing these immune cells (Ivanov et al., 2021). A series of works using mouse RAW 264.7 macrophage cell line (Escobar et al., 2013), MDMs (Ortiz et al., 2015), as well as dendritic cells (Zhu et al., 2012) has shown that gonococci induce tolerogenic phenotype in macrophages and dendritic cells so that they cannot induce the proliferation of T cells. More specifically, the role of different receptors on human dendritic cells, which included C-type lectins MGL and DC-SIGN, in the interaction with *N. gonorrhoeae* and induction of T cell response has been addressed using human monocyte-derived dendritic cells (van Vliet et al., 2009). Additionally, MDMs were used to demonstrate inflammatory pyroptosis induced by *N. gonorrhoeae* (Ritter and Genco, 2018), and human dendritic cells were used in studies of the connection between gonococcal and HIV-1 infection (Zhang et al., 2005). Lymphocyte models include the usage of chicken DT40 B cells, Jurkat cell line, and primary mouse and human lymphocytes (**Table 3**). The results stemming from these studies mostly pointed in the direction of gonococcal suppression of T cell proliferation and B cell humoral response (Boulton and Gray-Owen, 2002; Pantelic et al., 2005; Lee et al., 2007; Lee et al., 2008; Liu et al., 2012). Gonococcal interaction with memory B cells, which results in a broad, T cell-independent immunoglobulin response (So et al., 2012) has also been recorded. Regarding immune cells, the findings obtained with cell lines were often confirmed using primary cells, which was necessary to validate the results. However, the availability of primary immune cells is greater than the one of primary epithelial cells. They are therefore more often used in modeling of the gonococcal interaction with the host immune system, their disadvantage being donor variability.

Although primary cells enable us to better mimic the site of infection, their application has been limited due to their short life span, low proliferation rate, cross-contamination during cell isolation, and heterogeneity (Unger et al., 2002; Quillin and Seifert, 2018). Another source of primary cells could be induced pluripotent stem cells (iPSCs). They have already been used to study the related *Neisseria* species, *N. meningitidis*, and have proved useful in providing novel insights into meningococcal pathogenesis (Martins Gomes et al., 2019). All in all, the monoculture (2D) cell models provide the basic characteristics of the site of infection and are useful for addressing certain questions. They, however, cannot fully replicate the cellular complexity of a 3D model (Duval et al., 2017; Hoarau-Véchot et al., 2018).

## *In Vitro* 3D Tissue Models

### Artificial Scaffold

#### Intestinal and Urogenital Models

The transition from 2D to 3D cell culture techniques is a crucial step to obtain physiologically relevant tissue models for infection research. The advancements in tissue engineering and bioengineering enabled the development of numerous novel *in vitro* models of human organs, which can be applied in the field of infectious disease research too (Mills and Estes, 2016; DeCicco RePass et al., 2017).

Transwell^®^ inserts are widely used to culture columnar epithelial cells for studying the mechanism of pathogen transmigration through the polarized epithelial monolayer (**Table 4**). In 1998, human colorectal carcinoma epithelial cells (T84) were cultured on Transwell^®^ inserts to investigate the role of Opa binding to CD66 receptors in the transcellular traversal of *N. gonorrhoeae* (Wang et al., 1998). In the same year, HEC-1-B cells were seeded on collagen-coated Transwell^®^-COL membranes to study the role of pilus phase variation in *N. gonorrhoeae* transmigration through the epithelial layer (Ilver et al., 1998). The T84 monoculture model was also employed to investigate the role of the cytoskeleton and motor proteins in the transcytosis of *N. gonorrhoeae* (Wang et al., 2008). HEC-1-B and T84 cells on Transwell^®^ inserts were used as well to study the interaction of gonococci with polarized cells and these studies showed that gonococci weaken the apical junction and polarity of epithelial cells by activating EGFR, which facilitates their transmigration (Edwards et al., 2013). In a more recent study, the role of folliculin in controlling the intracellular survival and trans-epithelial passage of *N. gonorrhoeae* was shown using renal carcinoma epithelial cells (UOK 257) grown on Transwell^®^ inserts (Yang et al., 2020). Therefore, such models allow monitoring bacterial interaction with polarized cells, which is not possible when cells are cultivated in 2D.

**Table 4:** Selected 3D *in vitro* models used for studying *N. gonorrhoeae* infection.

Cells/Tissues	Platform	Key Findings	Reference
Fallopian tube organ cultures	*Ex vivo* model	Successful long-term infection of organ culture with *N. gonorrhoeae* outside the body.	(Carney and Taylor-Robinson, 1973)
Organ culture of the human fallopian tube	Human *ex vivo* model, perfusion bioreactor	Establishment of the perfusion-based system using human fallopian tubes for studying *N. gonorrhoeae* infection.	(Ward et al., 1974)
Fallopian tube organ cultures	*Ex vivo* model	Attachment of *N. gonorrhoeae* and the resulting damage of the oviduct mucosa.	(Johnson et al., 1977) (McGee et al., 1981)
The human cornea in organ culture	*Ex vivo* model	Thinning of cornea upon infection with gonococci.	(Tjia et al., 1988)
Distal ureters, human	*Ex vivo* model	Studying the mechanisms of colonization and invasion of *N. gonorrhoeae.*	(Mosleh et al., 1997
T84 cell line	Transwell^®^ insert	Traversal of polarized epithelium by *N. gonorrhoeae*.	(Merz et al., 1996)
T84 cell line	Transwell^®^ insert	The role of Opa binding to CD66 receptors in the transcellular traversal of gonococci.	(Wang et al., 1998)
Primary human endo- and ectocervical cells	Transwell^®^ insert	*N. gonorrhoeae* can invade endo/ectocervix cells and induce cytoskeletal rearrangements.	(Edwards et al., 2000)
Human endometrium	*Ex vivo* model	*N. gonorrhoeae* attach to cilia of endometrial cells.	(Timmerman et al., 2005)
T84 and HEC-1-B cell lines	Transwell^®^ insert	*N. gonorrhoeae* breaches the apical junction of polarized epithelial cells for transmigration by activating EGFR.	(Edwards et al., 2013)
*Ex vivo* porcine vaginal mucosa	*Ex vivo* model on Transwell^®^ insert	Interaction of commensal vaginal microbes with *N. gonorrhoeae*; *N. gonorrhoeae* grows in the pH 5.5 induced by lactic acid.	(Breshears et al., 2015)
HEC-1-A cell line	Rotating wall vessel bioreactor	A bioreactor model was developed for studying *N. gonorrhoeae* infection under dynamic conditions.	(Łaniewski et al., 2017)
Human endocervix, T84	Tissue explants, Transwell^®^ insert	*N. gonorrhoeae* induces non-muscle myosin II-mediated epithelial exfoliation.	(Wang et al., 2017b)
End1 cells, PMNs	Transwell^®^ insert (co-culture)	Neutrophil transmigration is dependent on *N. gonorrhoeae* contact with the epithelium.	(Stevens et al., 2018)
SV-HUC-1, HEC-1-B, T84, dermal fibroblasts	SIS scaffold-based 3D co-culture tissue model	Development of three novel human 3D tissue co-culture models based on SIS scaffold for studying gonococcal infection.	(Heydarian et al., 2019)
Cervix, HEC-1-B, T84 cell lines	Tissue explants, Transwell^®^ insert	Properties of cervix epithelial cells and pathogen surface molecules in infectivity of *N. gonorrhoeae.*	(Yu et al., 2019)
Excised bovine cornea	*Ex vivo* model	Establishment of *in vitro* eye model for studying *N. gonorrhoeae* infection.	(Churchward and Snyder, 2019)
UOK257 cell line	Transwell^®^ insert	The importance of folliculin in *N. gonorrhoeae* infection.	(Yang et al., 2020)
T84/fibroblasts/HUVEC/PMNs	SIS scaffold-based 3D co-culture tissue model, perfusion bioreactor	A model with epithelial, fibroblasts, endothelial cells, and neutrophils using a perfusion bioreactor.	(Heydarian et al., 2021)

Apart from cell lines, primary cells can be cultured in 2D for several passages and seeded on Transwell^®^ inserts for the generation of 3D models. This was shown for primary endometrial cells (Timmerman et al., 2005) as well as endo- and ectocervical cells (Edwards et al., 2000). Combining Transwell^®^ culture systems with organoid technology for *N. gonorrhoeae* research could allow a long-term expansion of primary cells due to specialized culture conditions and their subsequent usage in 3D Transwell^®^ models. Until now, methods for long-term culture of many of the epithelia relevant during *N. gonorrhoeae* infection have been established. These include ecto- and endocervical organoids (Chumduri et al., 2021; Lõhmussaar et al., 2021), endometrial organoids (Boretto et al., 2017; Turco et al., 2017), fallopian tube organoids (Kessler et al., 2015), intestinal organoids (Sato et al., 2009), as well as corneal organoids (Foster et al., 2017). Here, it is worth mentioning the multi-cellular model, containing stromal cells and organoid-derived epithelial cells of the endometrium on an artificial porous collagen scaffold, which produced polarized, hormone-responsive endometrial tissue models (Abbas et al., 2020). This is a fine example of how primary cells obtained by organoid cultivation can be transferred onto a scaffold, producing models of higher complexity, which can be used for various purposes, including infection research.

#### Corneal Models

Another common site of *N. gonorrhoeae* infection is the human cornea (Fransen and Klauss, 1988), for which different 3D models have been developed including cell lines as well as primary cells (Shiju et al., 2020). Some of the models were established using cells derived from pigs (Reichl and Müller-Goymann, 2003), cows (Churchward and Snyder, 2019), or rabbits (Drell et al., 1945). The latter was shown to mimic the natural features of *Pseudomonas aeruginosa* infection, considering that this pathogen can only invade the cornea connective tissue after prior corneal injury (Alarcon et al., 2009). However, since *N. gonorrhoeae* is a human-specific pathogen, usage of animal cells can pose a problem and there is a need for human cell-derived models.

The established human models include monocultures using only epithelial cells on Transwell^®^ inserts (Karamichos et al., 2012; Rajaiya et al., 2015) or the generation of corneal stromal equivalents (Ghezzi et al., 2017; Isaacson et al., 2018; Priyadarsini et al., 2018), as well as more complex models including co-culture of epithelial, stromal, or endothelial cells together (Zhang et al., 2017; Hutcheon et al., 2019). Even cultures including nerve cells have been developed (Wang et al., 2017c; Sharif et al., 2018). However, these models were not made with infection research in mind. Instead, diverse efforts were taken to replace the *in vivo* Draize test of eye irritation (Lotz et al., 2016; Lotz et al., 2018), generate 3D corneal models to use them as grafts in transplant medicine (Sharif et al., 2018), and for investigating specific pathologic conditions such as Keratoconus (Karamichos et al., 2012) or dry-eye disease (Park et al., 2018; Kaluzhny et al., 2020). On the other hand, adaptation and usage of these already-existing models in gonococcal research might provide new insights into the pathology of eye infection with *N. gonorrhoeae*.

#### Models Introducing Immune Cells

The female reproductive tract is comprised of layers of epithelial, stromal, and endothelial cells, providing barriers against pathogen invasion (Reynolds et al., 1992; Wira et al., 2005). Moreover, the presence of recruited immune cells during the pathogen challenge is an important feature of the female reproductive system (Wira et al., 2005). To study the interaction of *N. gonorrhoeae* with the cells of the immune system, several approaches have been attempted so far. Cell-free Transwell^®^ insert membranes were used to study the trafficking of *N. gonorrhoeae* outer membrane vesicles towards the bone marrow-derived macrophages seeded on the coverslip (Deo et al., 2018). One of the previous reports introduced the usage of a microfluidic device to quantitatively measure the 3D transmigration of neutrophils during an inflammatory reaction (Han et al., 2012). Transwell^®^ co-culture model of neutrophil-epithelial cells offered the opportunity to study the immune cell transmigration across the polarized endocervical (End1) cells in response to *N. gonorrhoeae* (Stevens et al., 2018). Traditionally, *in vitro* models based on Transwell^®^ and Dunn chambers that were used to study neutrophils consist of a well-in-well system. In these, an endothelial layer is formed on the membrane in the top well, followed by an introduction of inflammatory signals into the bottom well. With the addition of neutrophils to the top well, one can quantify the neutrophil migration to the bottom well. These models allow only end point analysis, and to monitor the real-time neutrophil migration and response more complex models are needed (Richardson et al., 2021).

### Decellularized Scaffold

Natural-based scaffolds such as decellularized tissues derived from heart valves, liver, blood vessel, nerves, skin, skeletal muscle, lung, and intestine are currently being investigated for the generation of *in vitro* and *ex vivo* models (Badylak, 2007; Steinke et al., 2014; Doryab et al., 2017; Massie et al., 2017; Wang et al., 2017a; Bölükbas et al., 2019). Acellular scaffolds contain key proteins of the ECM such as collagen, fibronectin, laminin, and in the case of porcine small intestinal scaffold (SIS), provide a mesh with interconnected pores, which offers suitable conditions for cell proliferation and differentiation (Gilbert et al., 2006; Shi and Ronfard, 2013; Wang et al., 2020). Recently, we have established three different co-culture models of epithelial cells and fibroblasts based on T84, HEC-1-B, and male uroepithelial cells (SV-HUC-1) using the SIS scaffold as support. We aimed at mimicking functional and morphological features of the site of the gonococcal infection in the human body, recapitulating both cell-cell and cell-matrix interactions. Investigations of the host cell-pathogen interaction using various bacterial strains and derivatives showed that the established tissue models based on the decellularized SIS scaffold are more resilient to infection, as well as that they support bacterial growth, enabling a longer observation time of up to six days. This makes such models suitable for long-term studies of infection and in this aspect superior to the commercial Transwell^®^ models (Heydarian et al., 2019).

Another level of complexity of the models is introduced by culturing the cells under conditions to which they are subjected in their natural environment. Bioreactors provide well-controlled cell culture platforms for supporting cell growth under dynamic culture conditions (Bancroft et al., 2003; Odeleye et al., 2020). In 1974, Ward *et al.* showed that cilia movements can block the *N. gonorrhoeae* attachment to cell surface using a perfusion-based bioreactor system (Ward et al., 1974). In this study, fallopian tubes derived from the patients were placed inside a perfusion system, where organ culture medium was perfused at the rate of 6 mL/h, followed by 30-60 minutes of circulation of *N. gonorrhoeae*-containing medium into the whole organ, to study the ability of *N. gonorrhoeae* to attach to and invade into the fallopian tube cells (Ward et al., 1974). In 2017, Łaniewski *et al.* used a rotating wall vessel (RWV) bioreactor to study the colonization of the endometrium by *N. gonorrhoeae*. They showed that the infection with *N. gonorrhoeae* significantly induced expression of proinflammatory mediators, causing ultrastructural alteration of the epithelial cells (Łaniewski et al., 2017), which corresponds to the clinical findings and qualifies this model for gonococcal infection research. We also utilized a perfusion-based bioreactor system to mimic the blood flow in a triple co-culture tissue model of T84, dermal fibroblasts, and human umbilical vein endothelial cells (HUVECs) for *N. gonorrhoeae* infection. The culture medium was circulated (perfusion rate of 0.5 mL/min) through the apical part (epithelial cells) and the isolated human neutrophils were delivered to the endothelial cells in the basal chamber using a perfusion rate of 2.5 mL/min. The perfusion-based bioreactor provided the opportunity to study the reverse transmigration of neutrophils, which is not possible under static culture conditions (Heydarian et al., 2021).

Moreover, *ex vivo* culture of explants, such as those from fallopian tubes (Johnson et al., 1977; McGee et al., 1981), kidney (Mosleh et al., 1997), vaginal mucosa (Breshears et al., 2015), and endocervix (Wang et al., 2017b) has been used in the *N. gonorrhoeae* research. The use of organ culture offers advantages by filling the gap between primary cell culture and *in vivo* conditions for answering questions related to microbiota, cell structures, such as cilia, or tissue exfoliation during infection. However, *ex vivo* culture models suffer from donor to donor variability (Grivel and Margolis, 2009) and depend on the availability of tissues. These issues can be circumvented by the generation of 3D tissue models of high complexity, containing all cell types relevant for the infection.

In summary, 3D tissue systems provide a more realistic environment, which more faithfully mimics the site of infection in comparison to 2D cell culture models. They are also more stable and with a longer cell lifespan, which is beneficial when studying long-term infection. However, certain challenges still need attention, such as batch-to-batch variability when using biological scaffolds, transparency of the tissues, and limited depth of microscopy (Antoni et al., 2015). The advantages of using cell lines when generating 3D tissue models lie in the availability of the cells, which is coupled to the option of generating a larger number of models with lower variability. However, not all cell lines enable the generation of biomimetic tissue models, or the models generated lack certain aspects of native tissues, such as cilia, glycogen granules, or mucus. Here, primary cells and explants can be a better option, despite the already mentioned problems of the availability of the tissues and donor variability. Finally, the model of choice depends not only on the technical aspects but also on the scientific questions asked.

## Future Direction

Many efforts have been undertaken to develop a suitable model for studying *N. gonorrhoeae* (**Figure 1** and **Tables 1**-**4**). Progress in the development of animal models and *in vitro* tissue models from simple 2D monoculture to complex 3D co-culture models has helped us to further our understanding of the pathogenesis of *N. gonorrhoeae*. Since the cells cannot form a multi-dimensional structure in 2D cell culture, 3D cell culture has emerged as an alternative to improve the cell microenvironment. Driven by the drawbacks of the animal models such as the lack of human receptors and anatomical differences, transgenic and hormone-treated animal models have been developed. Even though the *ex vivo* and *in vitro* models of tissues showed promising results in recapitulating the main characteristics of the site of infection in humans, each of these models has also its disadvantages. The transition between rather simple 2D monoculture to complex biomimetic co-culture and triple co-culture tissue models can assist us in further comprehending the crosstalk between epithelial cells and immune cells during gonococcal infection. In addition to the epithelial and immune cells, stromal cells have been shown to contribute to the growth of epithelial cells as well as to the tissue response to hormones (Arnold et al., 2001; Bläuer et al., 2005), indicating the importance of co-culturing models of epithelial cells with fibroblasts. Moreover, fibroblasts appeared to be a niche for *N. gonorrhoeae* internalization (Waitkins and Flynn, 1973) and were shown to play a crucial role in the long-term infection of the 3D co-culture model of epithelial/fibroblast cells (Heydarian et al., 2019), which further emphasizes the necessity of their presence when modeling tissues.

Apart from the cell type and origin, bioreactors and microfluidic platforms also play a key role in the increase of the degree of biomimicry of the tissue models, from static to dynamic. Until now, most of the investigations in the field of *Neisseria* infection have been performed under static conditions, which ignores the dynamics of the natural environment where infection takes place, such as the urethra of men, or blood stream. Recently, a microfluidic culture model of the human reproductive tract has been introduced, which simulated the endocrine loops between organ modules of the ovary, fallopian tube, uterus, cervix, and liver (Xiao et al., 2017). In addition, a variety of microfluidic systems have been developed to study neutrophil migration, neutrophil extracellular traps, and reactive oxygen species production (Agrawal et al., 2008; Boneschansker et al., 2014; Moussavi-Harami et al., 2016; Yang et al., 2017). Microfluidic devices work well for studying the immune response as they can be customized, require fewer reagents and material, and are ideal for working with primary human cells. One can control the spatiotemporal presentation of signaling molecules and the pathogen added to the system, making them applicable for studying complex interactions. Current developments in the microfluidics models allow one to incorporate live and intact pathogens into the models. This not only increases the significance of neutrophil response but also allows studying the direct interaction between neutrophils and a pathogen. The major advantage of microfluidics devices lies in their ability to allow single-cell analysis amidst neutrophil heterogeneity. Continuous development of such devices will allow extensive insights into signals controlling neutrophil function (Richardson et al., 2021).

Further developments of the tissue models would also include the introduction of the microbiome, which is present in various parts of the human female reproductive system (Chen et al., 2017). A correlation between the microbiome, menstrual cycle, and immune responses of the genital tract has been reported (Rönnqvist et al., 2006; Mirmonsef et al., 2011; Chen et al., 2017). Exploring host-microbiome interactions during the infection would help reveal the protective role of commensal microorganisms against the infection.

Recently developed 3D cell culture models such as organoids of the fallopian tube (Kessler et al., 2015), human endometrium (Boretto et al., 2017; Turco et al., 2017), and cervix (Maru et al., 2020; Chumduri et al., 2021) have opened new avenues in the field of investigations of the pathology of the female reproductive tract, including infection research (Alzamil et al., 2021). Human fallopian tube organoids have been successfully used for studying *Chlamydia trachomatis* infection (Kessler et al., 2019). Many organoids, however, are formed with the epithelial/luminal surface on the interior, which requires the infection to be performed either by microinjection, by reversion of the organoids, or by disruption of the organoids with reseeding after infection [reviewed in (Aguilar et al., 2021)]. In addition to being used as infection models, organoids can also be a source of primary cells for scaffold-based 3D tissue models, which helps to overcome the problems coupled with the infection of the organoids. This will allow us to take one more step towards obtaining high-fidelity tools for studying gonococcal infection under close to natural conditions. Considering the latest advancements in the generation and development of 3D tissue models, we can say that we are on a good way to achieving that goal.

## Author Contributions

All authors have written parts of the manuscript. MH, RR, and VK-P have generated tables and the figure. All authors contributed to the article and approved the submitted version.

## Funding

This work was funded by the Deutsche Forschungsgemeinschaft (DFG) GRK 2157 “3D Tissue Models for Studying Microbial Infections by Human Pathogens” to VK-P. This publication was funded by the German Research Foundation (DFG) and the University of Wuerzburg in the funding program Open Access Publishing.

## Conflict of Interest

The authors declare that the research was conducted in the absence of any commercial or financial relationships that could be construed as a potential conflict of interest.

## Publisher’s Note

All claims expressed in this article are solely those of the authors and do not necessarily represent those of their affiliated organizations, or those of the publisher, the editors and the reviewers. Any product that may be evaluated in this article, or claim that may be made by its manufacturer, is not guaranteed or endorsed by the publisher.
